# Marginalisation and distrust in the context of the COVID-19 vaccination programme: experiences of communities in a northern UK city region

**DOI:** 10.1186/s12889-024-18308-0

**Published:** 2024-03-19

**Authors:** Stephanie Gillibrand, Dharmi Kapadia, Ruth Watkinson, Basma Issa, Charles Kwaku-Odoi, Caroline Sanders

**Affiliations:** 1 Centre for Primary Care & Health Services Research, School of Health Sciences, Faculty of Biology Medicine & Health, The University of Manchester, Greater Manchester, UK; 2 NIHR Applied Research Collaboration for Greater Manchester, Greater Manchester, UK; 3 School of Social Sciences, University of Manchester, Greater Manchester, UK; 4 Independent public contributor, Greater Manchester, UK; 5 Caribbean and African Health Network, Greater Manchester, UK

**Keywords:** Vaccines, COVID-19, Marginalisation, Inequalities, Distrust

## Abstract

**Background:**

There are clear inequalities in COVID − 19 vaccination rates amongst marginalised groups, with lower rates for some minoritised ethnic and religious groups, younger people, those living in more deprived areas, and with lower socio-economic status. Existing research focuses on psychological and socio-economic factors that influence vaccine uptake and does not explore broader social and historical contexts. Understanding inequalities in COVID-19 vaccine uptake requires a critical examination of the drivers of, and barriers to, vaccination.

**Methods:**

We present findings from a co-designed qualitative research study undertaken during the COVID-19 pandemic. Focus groups and interviews were used to examine the context underpinning responses to the COVID-19 vaccination in Greater Manchester, particularly focussing on experiences of marginalisation. Thematic framework analysis was used to analyse the data.

**Results:**

We found that the public’s responses to the COVID-19 vaccination programme are intertwined with a longstanding history of institutional distrust and disenfranchisement, resulting from experiences of marginalisation and social inequalities. This was exacerbated further by the disproportionate impacts of the COVID-19 pandemic on minoritised ethnic groups, younger people, and those with existing health conditions.

**Conclusions:**

Histories of structural inequalities experienced by minoritised groups invoked feelings of suspicion and scepticism at the motivations of the agencies behind the vaccination rollout. This highlights the need for a contextualised analysis of attitudes to vaccines, considering pre-existing inequalities, which may be especially relevant for conceptualising public responses to the vaccination programme. Finally, our study shows the important ways in which public (dis)trust can impact public health policies. We recommend this should be incorporated into responses to future public health crises.

## Background

The national COVID-19 vaccination programme has been championed as imperative to combating the coronavirus and ensuing global pandemic. Overall, the programme has achieved high rates of vaccinations in the UK; by July 2022, 93% of the UK population (aged 12+) had received their first dose, and 69% had received a booster [1]. However, there are clear inequalities in rates amongst marginalised groups, with lower rates for some minoritised ethnic and religious groups, younger people, those living in more deprived areas, those with lower socio-economic status including lower individual education levels and unemployment, and those with low English-language proficiency [2–5]. In response, there has been a significant amount of research aiming to explore attitudes to the COVID-19 vaccine, including estimating the likelihood of uptake, building on previous work attempting to understand the complexities around vaccination motivations [6]. However, existing research in this space tends to focus on psychological and socio-economic factors that influence individuals’ vaccine uptake [7–10], and does not explore broader social and historical contexts or people’s experiences of marginalisation. Particularly neglected are discriminatory experiences rooted in institutional and structural systems within society which may impact current views towards state-sponsored public health drives such as the COVID-19 vaccination programme.

This paper reports on a co-designed qualitative study investigating experiences of local communities and public responses to the COVID-19 vaccines within a northern UK city region (Greater Manchester) during the COVID-19 pandemic. It supplements existing analysis from Watkinson et al., which finds that the introduction of the COVID-19 vaccination rollout exacerbated pre-existing inequalities in routine vaccinations in the Greater Manchester region, with evidence of wider inequalities amongst minoritised ethnic groups [11]. The findings from Watkinson et al. are particularly significant given that inequalities in uptake in the region are concentrated amongst marginalised groups most vulnerable to the virus (i.e. those living in the most socially deprived areas and older age groups). The study suggests that broader and contextual-based factors may be at play, such as public distrust and practical access barriers to the COVID-19 vaccines.

This paper interrogates some of these broader contextual factors through an exploration of attitudes to the COVID-19 vaccination programme amongst marginalised groups. We draw on findings from semi-structured interviews and focus groups to explore how historical experiences of marginalisation have impacted responses to the vaccination programme in Greater Manchester.

From the early stages of the COVID-19 pandemic there has been an upsurge in academic literature on attitudes to vaccines, with a view to gauging the acceptability of COVID-19 vaccines amongst different groups. These studies assessed vaccination intentions, finding that, whilst many people intended to get vaccinated, substantial proportions of people did not intend to get vaccinated because of a fear of side effects, reliability and efficacy [12], and a lesser perception of harm caused by the virus [13, 14]. Other reasons associated with lower vaccination intention include safety concerns, lack of institutional trust, confusing and conflicting information [15] and conspiracy beliefs [16]. However, much of this research has focused on specific populations, e.g. healthcare workers [15, 17]) [18–20] and other key workers [21]. Further, much of this research was undertaken prior to when the majority of the eligible population were offered their first vaccine dose during the national rollout (see, for example, [12, 13, 22, 23], and therefore only provides a snapshot of views on this complex topic. Indeed, a longitudinal study (February-March 2020– March 2021) found that people’s views towards COVID-19 vaccines changed during the course of the pandemic, with higher perceived risks of vaccination in the later period (March 2021) following increased media coverage, especially of side effects to the AstraZeneca vaccine [24].

Moreover, most published research has tended to be predominantly quantitative. These studies have assessed associations between socio-demographic factors and vaccination attitudes, finding that negative attitudes towards vaccination are associated with: being younger in age (16–24 year olds), being female, belonging to a non-White ethnic group, lower levels of education, and lower household income [7–10, 16]. These studies identify some key factors surrounding vaccination attitudes and subsequent uptake, however, fail to explain why associated factors are relevant to individual and collective responses to the COVID-19 vaccination programme.

Institutional trust and trust in medical professionals and experts are important indicators of vaccine uptake [25], and mistrust towards government and scientists is a prominent factor associated with not being vaccinated [7, 26, 27]. For example, recent evidence from the UK suggests people from minoritised ethnic groups who had experienced racial discrimination in a medical setting were less likely to receive a vaccine than those who had not. Moreover, these effects were mediated by low levels of trust in the health system surrounding the handling of the pandemic [28]. Further, racism has been shown to be a significant driver in ethnic inequalities of receptive attitudes to the COVID-19 vaccine [29]. This research highlights that broader contextual factors play a significant part in explaining attitudes to the COVID-19 vaccine [30].

### Marginalisation & (dis)trust in the context of the pandemic

The pandemic has disproportionately impacted marginalised groups, such as minoritised ethnic groups, people living in deprived areas, people with poor health and long-term health conditions and people with learning disabilities [31, 32]. Experiences of marginalisation stem from social inequalities (e.g. on the basis of income, social class, occupation, education level, housing, region, ethnicity etc.), leading to differential health outcomes experienced on the basis of these social inequities [33, 34], whereby these groups may experience a disproportionate prevalence of health conditions and worse health outcomes and can therefore be considered as marginalised [35].

Experiences of marginalisation are compounded for those who belong to more than one marginalised group [36]. For instance, rates of poverty are higher amongst minoritised ethnic populations [37], impacting outcomes such as life expectancy, which is lowest amongst Pakistani and Bangladeshi people, and is associated with area-based socio-economic factors [33, 38]. As such, assessing the context of marginalisation experienced through pre-existing inequalities may be especially relevant for assessing responses to the COVID-19 vaccination programme, where the pandemic has (re)produced long-standing social, economic and health-based inequalities [39, 40]. Examples include the disproportionately high COVID-19 mortality rates in the most deprived areas [41, 42], and amongst Black African, Pakistani and Black Caribbean groups compared to White British groups [43].

Distrust may be one of the mechanisms through which marginalised groups have poorer health outcomes [44–46]. Distrust may simply be defined as the absence of trust [47]. However, broader literature reflects the dynamic shaping of distrust, and the complexity of contributing factors incorporating specific grounds for institutional distrust, or the erosion of trust towards institutions, governments etc., altogether [48, 49]. For example, regional and income-based inequalities have been linked to individual level distrust towards institutions in the UK [50], and other prominent drivers of distrust include political dishonesty and untrustworthiness, a lack of accountability, betrayal of political promises, and failure to protect the most vulnerable in society [51]. Drivers of distrust may also stem from wider institutional factors and public policies contributing to unequal outcomes experienced by marginalised groups, including minoritised ethnic groups. For example, immigration policies (e.g., the ‘hostile’ environment) and national security policies (such as Prevent), explicitly target racialised populations, where such policies are rooted in racialised discrimination and prejudice [52]. Alongside this, some communities have faced increased racialised violence in the face of Brexit [53] and more recently during the pandemic [54], including institutionalised violence towards minoritised ethnic groups [55]. Consequently, distrust may be especially strong amongst socially disadvantaged, marginalised and minoritised ethnic groups, due to systemic and historical forms of oppression, inequality, and racial discrimination [56–58].

## Exploring responses to the COVID-19 vaccination programme in greater Manchester

During the pandemic, Greater Manchester experienced higher levels of mortality from COVID-19, higher case rates and greater impacts to productivity than the national average [39]. Overall, the North of England spent longer in lockdown and experienced adverse trends in poverty, education, employment and mental health for children and young people, exacerbated since the onset of the pandemic [39]. COVID-19 vaccines were first offered to the public in December 2020, and in Greater Manchester administered at hospital hubs, mass vaccination centres, and local vaccination services [59]. The aim of this research project was to explore public responses to the COVID-19 vaccination programme to understand how inequalities can be addressed, learning from the perspectives and experiences of under-served communities and key stakeholders.

Here, we draw on data from qualitative interviews and focus groups with 35 participants based in Greater Manchester (including, residents from local communities disproportionately impacted by the pandemic, community leaders, and health and care system stakeholders) to explore the public health and broader socio-political landscape, considering the social context and experiences of marginalisation prior to, and during, the COVID-19 pandemic.

## Methods

### Sampling & recruitment

The sampling strategy for the study prioritised marginalised groups that have been disproportionately affected by the COVID-19 pandemic in England. This included (but was not limited to) minoritised ethnic groups [42], young adults [60], and those with long-term physical and mental health conditions [61, 62]. The study explicitly targeted younger people as a seldom-heard group within research but ensured a balanced and inclusive age range of participants.

A combination of a purposive and snowballing sampling approach was used to recruit people via pre-established links and connections to community networks and key stakeholders to ensure the inclusion of specific seldom-heard groups. For example, people from African and Caribbean backgrounds were recruited via a charity that seeks to tackle health inequalities for Caribbean and African populations in England, and people from South Asian backgrounds were recruited via a mental health charity based in Rochdale. Collaborators from these organisations shared details of the study and an e-poster to mailing lists via email and word-of-mouth. Those with long-term health conditions were recruited via the Research for the Future^1^ database, in which details of the study were circulated via online mailing lists. Community leaders were recruited via links to Advisory Group members and through a purposive sampling of local religious organisations. Health and care system stakeholders were sampled via online circulation of the study via pre-existing contacts amongst the Greater Manchester Health and Social Care Partnership.

### Study design

The study utilised a participatory approach [63] throughout the design and implementation of the research project. The work was co-designed and co-produced with community partners via a Community Research Advisory Group (CRAG). Approaches to co-production vary, but we aimed to follow core values and principles of co-production as outlined by the National Institute for Health Research [64], including power-sharing and shared decision-making, building and maintaining relationships, and by including a diversity of skills and perspectives. Following this, the rationale and scope for this work grew from a related quantitatively-focused project looking at inequalities in COVID-19 vaccine uptake in Greater Manchester [11]. During the public engagement activities for the quantitative work, there was an identified need amongst public contributors for a more in-depth, qualitative workstream to explore the drivers behind inequalities in vaccine uptake. Those who had been involved in the public engagement for the quantitative work formed part of the CRAG for this qualitative workstream, and met regularly to inform the study design, analysis, and write-up of the findings. The CRAG was made up of three members, from local communities and diverse backgrounds, local community-based groups and specialist charities. Meetings were held every few months during the project lifespan and were held over Zoom as this was deemed the most accessible way to meet. The CRAG inputted on project scope and research questions, advised on sampling and recruitment approaches, and contributed to the analysis through anonymised data extracts and discussion of themes.

Qualitative data were collected using individual interviews and focus groups enabling a combination of personal case-studies, narratives, and discussion of shared experiences.

Participants involved in the study broadly relate to three participant groups: [1] *community residents;* were lay people (who belonged to one or more of the marginalised groups that were the focus of this study), recruited from diverse local communities who were disproportionately impacted by the pandemic, [2] *community leaders*; included community and religious leaders, members of local community VCSE (Voluntary, Community and Social Enterprise) organisations and smaller, informal community networks and groups from these local communities, and, [3] *health and care system stakeholders*; included local council workers and health and care system stakeholders organising the vaccination response in Clinical Commissiong Groups (CCGs) and GP Federations.

Four interviews (including one joint interview with colleagues in a GP Federation) were held with health and care system stakeholders and six interviews were held with community leaders. Twelve interviews were held with community resident participants, via Zoom or over the telephone (due to the pandemic). Three focus groups with community resident participants were held via Zoom. Two were undertaken with women from South Asian communities (four participants in each group). The third focus group was held with four participants with long-term health conditions. All interviews and focus group sessions were audio recorded and transcribed. Informed written consent was taken prior to the focus groups and interviews. The interviews and focus groups were facilitated by SG, with two focus groups (with South Asian women) co-facilitated by a CRAG member. This CRAG member also provided translation support (Punjabi) in one of the focus groups where required.

Within the interviews and focus groups, participants were encouraged to discuss areas that they felt were important to them within the broad areas of discussion, drawing on key themes identified in the public engagement work as important to wider discussions around the vaccination programme. This included experiences during the pandemic, attitudes to the COVID-19 vaccine, mistrust, engagement with communities etc. All participants completed a socio-demographic form, including details such as age, gender, ethnicity, and area (see Table 1). We did not collect data on individual level socio-economic measures in the demographic form, but area-level socio-economic information is represented at the locality level using the Index of Multiple Deprivation, where eight out of ten Greater Manchester localities were represented by participants. Of these localities, between 20 and 40% of the Lower-layer Super Output Areas (LSOAs) in Manchester, Oldham, Salford, Rochdale, Bolton and Tameside reside within the most deprived decile of deprivation [65].

The interviews and focus groups took place between September and November 2021.

**Table 1:** Sample characteristics (community residents, community leaders, health and care system stakeholder participants *n* = 35)

	Community resident participants (*n* = 24)	Community leaders (*n* = 6)	Health and care system stakeholders (*n* = 5)
**Gender**			
Male	7	3	1
Female	17	2	4
Prefer not to say	0	1	0
**Age**			
18–24	6	0	0
30–39	2	1	1
40–49	4	3	2
50–59	3	2	1
55–64	2	0	1
60–69	3	0	0
70–79	3	0	0
80+	1	0	0
**Ethnicity**			
African/African British	1	0	0
Arab	0	2	0
Bangladeshi/Bangladeshi British	1	0	0
Indian/Indian British	2	0	0
Caribbean/Caribbean British	2	0	0
Chinese	1	0	0
Jewish	0	2	0
Kashmiri	1	0	0
Pakistani/Pakistani British	6	0	0
White English, Welsh, Scottish, Northern Irish or British	10	2	5
**Areas in GM**			
Bolton	1	0	1
Bury	0	0	1
Manchester	7	2	0
Rochdale	7	0	1
Salford	4	3	2
Stockport	1	0	0
Tameside	1	0	0
Trafford	1	1	0
Other	2	0	0
**Total**	**24**	**6**	**5**

### Data analysis

Data were analysed using an adapted framework approach [66]. An initial list of themes was derived by the study team, based on the coding of four transcripts initially, and discussions with the CRAG members. This set of themes and sub-themes was used to code the remaining transcripts and evolved to include further inductively generated codes as analysis progressed, based on regular discussions within the project team.

The coding of the transcripts was shared between the research team, with one team member responsible for collating the coded transcripts into a charting framework of themes/sub-themes with illustrative example extracts from the transcripts. After this, these themes and sub-themes were sense-checked by the CRAG group and research study team, to collate and synthesise further where possible for a final iteration of the themes and sub-themes. The findings are presented in relation to three overarching themes: attitudes to the COVID-19 vaccines: benefits versus fear and distrust; disenfranchisement: historical and current experiences of inequalities amongst Greater Manchester communities; distrust: longstanding distrust towards the system, and we summarise with a final section which discusses the COVID-19 vaccine in a socio-political context.

### Ethical approval

This study was approved by University of Manchester Ethics Committee (Proportionate UREC) 24/06/21. Ref 2021-11646-19665.

## Results

In the findings below, we draw out details of each theme with exemplar quotes, to illustrate responses to the COVID-19 vaccination programme amongst respondents. We summarise with a section which situates the COVID-19 vaccine in a socio-political context.

### Attitudes to the COVID-19 vaccines: benefits versus fear and distrust

Responses demonstrated a range of views towards COVID-19 vaccines, spanning support for the vaccine to opposition or concern rooted in fear and distrust. Many positive responses amongst respondents centred the need to protect themselves and others from the virus. There was also a sense that getting vaccinated would help to achieve ‘getting back to normality’ in the context of social distancing restrictions and virus-related policy decisions.

For some community resident participants, the decision to get vaccinated was uncomplicated and straightforward. For others, potential benefits alleviated initial doubts.

*“I have really no hesitation towards it. I put my trust in the science that was behind it and the research that happened to go into it and stuff like that [ ] I was just like I was offered it, yeah, I’ll take it”* (community resident, male, 18–24, White/White British).

*“…as soon as I heard that the vaccine was on the cards, I’m like, yes, I knew straightaway. I didn’t even need to think about it”* (community resident, male, 50–54, White/White British).

*“…I feel the more people take the vaccine, they’re more likely that the virus can be controlled and then less people will die [ ]At first, I doubted it because, you know, I just feel like it’s insane that the vaccine can be produced [ ] in such a short time…at first, I just thought, is this vaccine even going to work [ ] afterwards, my view changed, because some of my friends and also, like my friend’s parents, they got the vaccine*” (community resident, female, 18–24, Chinese).

For many other community resident participants however, significant competing antagonisms were described, surrounding inter-personal and political pressure to receive the vaccine, alongside factors such as fear, safety concerns, and a lack of knowledge and sufficient information surrounding the vaccines themselves. This was highlighted across a range of community resident participants, across ethnic groups and different age cohorts of participants. For those with health conditions, concern was expressed over fear of the virus (therefore requiring protection from the vaccination) at odds with fears of side effects of the vaccine exacerbating pre-existing health conditions. In particular, some female community residents with long-term health conditions highlighted concerns towards the vaccine.

“*This hadn’t been trialled long enough and I didn’t want to risk having it but because they’d said it will protect people who are vulnerable, that’s how it was sold as, that it will protect people who are vulnerable, I had it myself*” (community resident, female, 55–64, Indian).

*“I at first was quite fearful to be honest of having it…I was more fearful that it was going to affect my health detrimentally basically”* (community resident, female, 40–44, White British).

This extended to concern towards family members with health conditions and a lack of information about how the vaccine may interact with certain conditions.

*“I’m pro-vaccine, but I’m quite worried about my son with his heart condition…I know there’s been research done, but it can affect the heart…And for me I’m thinking gosh, if that sets something off in his heart…then it’s just quite frightening, really”* (community resident, female, 40–44, White British).

Some participants highlighted notions of duty, morality and responsibility intertwined with their personal vaccination attitudes.

*“I feel like it’s the right thing to do, because I think we should all be pitching in and doing this together…to me, there’s no reason to not trust people telling us to get it.* " (community resident, male, 30–34, White British).

One community resident who was a community vaccinator observed that younger groups were more fearful of potentially harmful side effects of the vaccine (such as causing infertility and misinformation about vaccines containing microchips), compared to older groups. Confusion surrounding misinformation was also echoed by some younger community participants:

*“Sure, so I haven’t had the vaccine yet. Mainly because, obviously information, I don’t know all the information about it, of course. And you obviously see all this stuff on social media and I don’t really like listening to social media like that. Like I see one post saying, it has A, B and C and it has this side effect. But I see another post saying, oh but this has A, B and C and certain side effects. So me, I don’t know. And also there is also that speculation that it is still undergoing testing. So in a year or two you’ll have to get a new vaccine, even if you do get this one. So me personally, I feel like if I don’t know everything about it, I wouldn’t want to take it just yet”* (community resident, male, 18–24, Pakistani).

Some community resident and community leader participants, from Indian and Pakistani backgrounds, across different age cohorts, reported feeling conflicted about the decision to get vaccinated or not. This was also the case for some with pre-existing health conditions, from White, Indian and Pakistani backgrounds, citing safety concerns. Some expressed regret at having had the vaccine, and that, retrospectively, they were uncomfortable with their decision to get vaccinated.

“*I would say, in my heart, I’m not fully settled with being fully vaccinated…I sometimes think I shouldn’t have got it so soon, maybe I should have given it a couple of months*” (community resident, female, 18–24, Pakistani).

Alongside vaccine safety concerns, was a sense amongst some female participants with long-term conditions and from some women from South Asian backgrounds of a general lack of accountability amidst the vaccine, where it was felt that sufficient protections were not in place.

“*in terms of the accountability. If, God forbid, if there was something to go wrong after you’ve had the Pfizer vaccination, or whichever vaccination you’d had, if there are any long-term concerns and, you know, effects because of that, it clearly states that you can’t hold anybody accountable. There’s nobody to, you know, nobody’s door to knock on…if anything was to happen, something went wrong, who’s door do I knock on, but nobody is taking the accountability for that”* (community leader, female, 45–49 Pakistani).

### Disenfranchisement: historical and current experiences of inequalities amongst greater Manchester communities

Comments about the COVID-19 vaccine made by community resident participants indicated that participants felt a high degree of disenfranchisement in the contemporary socio-political climate in England. These views were reflected upon about previous, historical experiences of deprivation, stigma, discriminatory practices and unfair social outcomes, which still resonated amongst current experiences, and was expressed across participant groups. This was often discussed in and around the context of the pandemic and the vaccination programme.

“*It’s a lifetime of experience, [], you’re waiting for the outcomes and nothing happens, yet some people are sleeping under the arches and others have got three or four houses. []…as you can tell, I’m not into injustices*” (community resident, female, 75–79, Black Caribbean).

These deep-rooted sentiments were reignited as direct ramifications of the witnessed actions, consequences, and impacts of the pandemic.

“*I think this pandemic really let us know how many cracks there are in the NHS, how underfunded the NHS is… yet MPs can get a rise in their wages, yet we can go to war with Tom, Dick, Harry and there’s always money for that, there’s always money to give tax breaks to millionaires and billionaires, but the key workers, the core people that run and make sure we’re healthy are so underfunded, so unappreciated that it’s laughable that we are a first world country and this is how we treat our healthcare workers”* (community resident, female, 45–49 years old, Kashmiri).

For some community resident participants, longer-term injustices were perceived to be further intensified during the COVID-19 pandemic, and links were made between long-term failings for underserved communities and the disproportionate impact of the pandemic.

“*You just can’t help but think if we as a BAME community had support in the first place maybe we wouldn’t be the vulnerable ones through COVID*.” (community resident, female, 18–24, Pakistani/Pakistani British).

“*Some people have made a lot of money out of it, and the rest of us die or suffer…*” (community resident, female, 75–79, Black Caribbean).

It was also highlighted by both community leader and community resident participants that the disproportionate impact of the pandemic on communities laid bare pre-existing observations that their communities had been left behind by decision-makers through entrenched inequalities. It was felt that the current pandemic emphasised previous failings for under-served communities and highlighted past experiences of discrimination and racism. This reproduced fear and suspicion amongst some minoritised ethnic groups towards the vaccination programme and pandemic response more widely, as identified by community residents and community leader participants. A faith leader described:

“*there’s always this undercurrent of mistrust of mainstream services…In the past there’s been institutional racism which made people very antagonistic towards mainstream services. Now, one would hope these days, that these days that’s much quieter, but, you know, ethnic minority communities have very long memories, and this kind of fear, it’s a fear also, it’s not just a mistrust, it’s a fear, is almost part of our DNA, it’s just inbred in so much about what we do and how we operate…the fact that our community was so heavily affected in the first few waves of the pandemic, will, if anything, exacerbate this, because people will say, well the mainstream services didn’t help us, they didn’t care for us*” (community leader, 55–64, Jewish).

For some community resident participants, namely South Asian and Chinese women, this overlapped with experiences of heightened racism during the pandemic.

*“as we’re walking the streets we get, it’s people like you that spread it, it’s people like you who aren’t educated enough, people like you shouldn’t live in this country…When I wear this I’m a target because apparently according to my Prime Minister I look like a letterbox. So, I’m already a target…”* (community resident, female, 45–49, Kashmiri).

*“…during the start of the pandemic [there was] lots of hate crime towards, like, Asian people []…I remember [when] I walk[ed] on the street, and a random guy just, like, said to his kids, see, this is the virus, and [that] just really hurt me at that time. So, I feel like, just more safer staying at home than going outside now.*.” (community resident, female, 18–24, Chinese).

For younger community resident participants, amongst different ethnic backgrounds, experiences during the pandemic intersected with social insecurity, including worklessness and the closure of educational settings. One participant, discussing circumstances of a peer within the context of the vaccination programme, highlighted:

*“The friend that doesn’t trust the government, that was a belief that stemmed before the pandemic, I guess. He’s very far left anyway with his political agendas and stuff. He was very anti government anyway. But I know it heightened for him because he was set to go in to become a fireman and then he had the submission revoked when the pandemic started because the pandemic had started, and it was kind of like his dream job. He’d waited ages to go through the process to become one and then they’re like okay, we can’t do it anymore, we’re taking away the place…he let his anger out towards the government for the reason of the pandemic, which I understood because I was also in the process of trying to look for a new career….So I did kind of share in that frustration a little bit where I was like this is going to stop me from progressing in my life”* (community resident, male, 18–24, White British).

### Distrust: longstanding distrust towards the system

It was clear that many community resident participants, especially those with long-term health conditions and family members with health conditions, did not trust the government to ensure their safety, and suspicions were raised about the motivations behind policy decisions throughout the pandemic. This was epitomised by perceived contradictory actions and messaging from the government, as well as misleading information around the COVID-19 vaccines and issues around accessibility of information, including language barriers. This was universally highlighted among participants across different ethnic backgrounds, ages and genders.

“*there’s the trust issue with this, how much they want us to know. They want us to go back to work, they want people back in offices, they want people back on trains and on buses, because that economically is important to the government*” (community resident, male, 60–64, White/White British).

“*…we do need to know more about what’s going on. They only tell us what they want us to hear”* (community resident, male, 55–64, White/White British).

For some female participants from Indian, Pakistani and Kashmiri backgrounds with long-term health conditions or family members with health conditions, this intersected and exacerbated fears towards the vaccine.

*“I just feel very let down by the government that had they been very… I feel they haven’t been honest all along and I think I’m not the only one who feels that. I think majority of the UK population feels that, that the government has never been very honest about anything to do with COVID*…*I can’t trust anything the government says. Because they’ll say one thing, then they’ll do a U-turn and say something else“* (community resident, female, 55–64, Indian).

Some community resident participants felt that the government had weaponised elements of the pandemic to gain public adherence to lockdown measures and the vaccination programme. Distrust was clear amongst female participants from some minoritised ethnic backgrounds, where this was linked to experiencing and witnessing entrenched social inequalities.

“*…the only way they can get under the armour of most people is by fearmongering, and fear is bigger than COVID…All I would like is to be told the truth, whatever it is. I don’t want lies wrapped up…[] Wash your hands, cover your face and protect the NHS. It’s their job…[]We have not been told the truth and we’ve been herded like sheep, those who die, die….[]I don’t think they’ve been honest with us at all…”* (community resident, female, 75–79, Caribbean/Caribbean British).

Distrust was explicitly highlighted by health and care system stakeholders and some community leader participants as a key factor impacting vaccine uptake. For instance, it was identified by community leaders, health and care system stakeholders and some community residents that the cohort of groups that were not getting vaccinated were those already disengaged with mainstream services, tending to be already marginalised groups that overlapped with those not getting the vaccine. Behind this, were identified power imbalances as a consequence of previous negative experiences for marginalised communities. A participant who works as part of and with a range of VCSE groups described:

*“when you’ve got structures like the council and the NHS, how they are perceived in the community; it’s like us and them… You know, particularly around councils, you know, they are trying to do outreach. But if you’re going on outreach in a very deprived community there is often the historical fear that these people take away our kids. And, actually, there’s a massive distrust there about accessing those services”* (community leader, female, 45–49, White British).

Distrust was explicitly articulated via the government’s handling of the pandemic by community residents and community leader participants, articulating this in terms of the communities they support, as well as suspicion towards motivations behind the vaccine rollout. This compounded attitudes of distrust surrounding the political drivers behind the vaccination programme.

“*quite a lot of patients wanted to know why they couldn’t see what’s been drawn up. I had to explain to them that the nurses keep the vaccine in a controlled area…But they still wanted to know why it wasn’t being drawn up in their sight”* (community resident, female, 55–64, Indian/Indian British).

Distrust among marginalised groups (including people living in deprived areas, socially vulnerable groups and certain religious communities) was emphasised by health and care system stakeholders also, who identified distrust as a consequence of previous negative experiences as related to vaccination uptake.

*“…I think it was a bit of distrust really in the wider Muslim community about past experiences and they weren’t clear whether it was acceptable from a cultural perspective, taking the vaccine, and there were some mixed messages there.”* (health and care system stakeholder, male, 40–44, White British).

Discussing the communities that they are part of, some community leader participants reflected on their communities’ perspectives. A faith leader described:

*“In ethnic minority communities, there’s always a certain suspicion towards science and scientific advances, until they’ve been absolutely proved, and especially vaccines, where you had this whole thing of the MMR vaccine, that it caused autism. So, there’s always this lingering suspicion about, you know, what’s really going on with vaccines?”* (community leader, 55–64, Jewish).

This was emphasised by a community leader participant who hosts an informal community network of local women.

*“Things to say that it will change your DNA for example, things about that children won’t have, you know, they would be infertile if they take the vaccine, that’s one of the main things that people [thought], ‘cause they don’t want more of us, so they’re vaccinating [us] not to have children*” (community leader, female, 50–54, Arab).

Others highlighted that it was not distrust in the vaccine per-say, but highlighted concerns around efficacy, perpetuating fears around effectiveness. A participant who runs a charity which supports local vulnerable communities described:

*“Sometimes they talk about, oh I know many people who got the vaccination and they got COVID again so what’s the effectiveness of this vaccination.*” (community leader, male, 40–44, Arab).

For some community resident participants, institutional distrust over concerns towards the vaccine’s safety and long and short-term side effects, epitomised and compounded long-standing fears and anxieties. This was rooted in historical experiences and trauma, alongside current experiences of side-effects to the COVID-19 vaccines, reflecting high levels of distrust, fear and suspicion, and for some participants, reiterating previous mistreatment of minoritised ethnic groups in scientific experimentation. This was inter-woven with experiences of personal ill-health or family members who had had bad health.

*“I’ve seen what vaccine damage does. I’m the youngest in my family. I had a younger sister, born completely normal, had a vaccine– I was too young at the time but I believe it’s whooping cough– and couldn’t talk, couldn’t stand, couldn’t hold anything, couldn’t hold her head. That’s what became of her. We were still fighting with it five years later when her lungs collapsed and she was in hospital and she died. We were still fighting about that.* " (community resident, female, 45–49, Kashmiri).

*“…I’ve had my first vaccination and I as an individual have seen what it’s done to me, how I am feeling now. Somebody who is always active, running around 24[/7],…I don’t feel myself. I’m getting body aches. I feel exhausted []…I’m very reluctant to take the second one because personally for me I’m feeling worse since I’ve had it.”* (community resident, female, 50–54, Kenyan/Kenyan British).

### The COVID-19 vaccine in a socio-political context

The context surrounding the COVID-19 vaccination rollout is embedded within broader sentiments of socio-political disenfranchisement and, amongst some participants, long-standing distrust. This culminated in the ‘perfect storm’ entangled with stigma and experiences of othering amongst some people from minoritised ethnic backgrounds. Specifically, some participants highlighted issues around a perceived lack of choice and a lack of personal autonomy surrounding the political pressure to receive the vaccine.

*“…forcing us. It’s not recommending now. Enforcement. If you don’t get it you cannot live your life in England now because of that vaccine.”* (community resident, female, 50–54, Pakistani/Pakistani British)/.

*“Now, the more that the government and the powers that be put those restrictions in place, the more we feel cornered to make that decision, which is not necessarily something that we want to do, but it restricts our freedom and mobility around the world…I have decided not to have the vaccine, not because I’m against vaccines, my children have had it, because that’s the only power I have. I’ve got no power. I feel powerless. We are powerless.”* (community resident, female, 45–49, Pakistani/ Pakistani British).

“*the pressure to take that vaccine has become so much because we don’t want to be blamed. It has no longer become a choice. It has become it’s you against them; if you’re not with us you’re against us. And that’s the pressure the BAME community is facing more and more…*“ (community resident, female, 45–49, Kashmiri).

A community leader participant who hosts an informal community network of local women described:

*“I think some of them are still there, even people who took the vaccine, some of them believe, probably, they would say, I was wrong in giving it to my child, but I had no option, I have to do it, and also everybody else had done it.” (*community leader, female, 50–54, Arab*).*

## Discussion

The findings presented here detail responses to the COVID-19 vaccination programme in the context of experiences of marginalisation. For many participants, the decision to get vaccinated was straightforward and uncomplicated, with clear reasons highlighted around personal and general public health benefits. Other participants, especially those with pre-existing health conditions or family members with health conditions, and including people from multiple ethnic groups (including Pakistani, Kashmiri, Indian, Chinese and White backgrounds), held a more conflicted stance on COVID-19 vaccination. This was often driven by a lack of faith in the efficacy and safety of the vaccine and a lack of information about the vaccine.

Conflicted views were often interlaced with anger towards public messaging about the vaccination, as well as established public health drives to boost uptake, which it was felt was built on features of divisive public messaging. For many, this indicated an attempt to force vaccine uptake, encroaching on principles of autonomy and personal choice, serving to reinforce unequally felt outcomes for marginalised groups. For many community participants, the decision to get vaccinated transcended reasons around personal health benefits, and became embroiled with tensions towards the vaccination rollout. Here, pushback against the vaccine is articulated via a sense of establishing boundaries against an oppressive system, defined by entrenched inequalities. Consequently, the choice to not have the vaccine, may, for some participants become almost a metaphor for political dissent against institutionalised failures for under-served communities who experience marginalisation.

Discussions surrounding the COVID-19 vaccine revealed widespread disenfranchisement amongst participants. This was expressed in more specific terms for women from minoritised ethnic backgrounds (specifically Pakistani, Indian, Kashmiri, and Caribbean participants), as articulated via a prominent legacy of historical, social and income-based inequalities. Such inequalities were viewed to be reproduced and compounded by the ensuing pandemic through disproportionate health-related outcomes, divisive public messaging and policy-measures. This was also articulated in the experiences of younger participants and those with pre-existing health conditions. This included experiences of worklessness and poor mental health in light of significant disruptions to everyday life, furlough, and the closure of work opportunities and educational settings. For some younger participants and participants from minoritised ethnic groups (Pakistani, Kashmiri and Chinese), this intersected with heightened experiences of racism experienced during the pandemic. Whilst this study includes participants from a range of minoritised ethnic groups, the common views and experiences associated with histories of marginalisation and pre-existing inequalities indicates the findings may be relevant for additional marginalised groups not represented in this research.

The intensified continuation of these inequalities, as witnessed and experienced by participants from groups most disproportionately impacted during the pandemic, sought to re-affirm pre-existing observations and experiences that marginalised communities are ‘left behind’ by policy and decision-makers prior to and during the pandemic. Here, the well-documented media and policy attention towards disproportionately high virus rates in the North West of England and amongst certain minoritised ethnic groups, alongside reporting of differential vaccination rates across different social and ethnic groups, reproduced feelings of othering, stigma and blame experienced amongst participants from minoritised ethnic groups (including Jewish, Pakistani/Pakistani British and Kashmiri participants). The combination of this and culturally insensitive public messaging and practices during the pandemic has only heightened longstanding and widespread disenfranchisement and has impacted broader responses to the vaccination programme amongst many participants. This reiterates findings from a recent study which found that reporting and framing (in the media and by politicians) of inequalities around the COVID-19 pandemic exacerbated feelings of stigma amongst minoritised ethnic groups, reinforcing othering and narratives of blame [67].

Distrust is a particularly prevalent theme across the research findings. Feelings of distrust were emphasised by a range of community residents and community leader participants via the perceived mishandling of the pandemic by the government and paradoxical government policy-responses to aspects of the pandemic, including U-turns and mixed public messaging.Participants with pre-existing family or personal health conditions highlighted a failure of the government’s response to protect the most vulnerable, and perceived that the economy was privileged over public health during the course of the pandemic. Multiple layers of distrust were articulated amongst many participants (including participants from African, Arab, Caribbean, Indian and Pakistani participant groups), as the intersectionalised consequence of deep-rooted institutional distrust resulting from past experiences and institutionalised oppressions. Elements of distrust appeared to be established prior to the pandemic, with distinctive elements having enhanced these sentiments during the pandemic, not least due to intensified episodes of racism experienced over the course of the pandemic. This echoes existing literature in which the links between distrust and healthcare and health outcomes [45, 68], especially amongst people from minoritised ethnic backgrounds [46, 69, 70] is well documented.

It follows that this has specific implications for the vaccination programme, where established and pre-existing fears stemming from historical experiences and social contexts may help to explain entrenched distrust towards institutionally-backed vaccination drives amongst groups who experience marginalisation, on the basis of ethnicity, age, health conditions, or the combination thereof. Indeed, it was also recognised by health and care system stakeholder participants that institutional distrust was a prevalent factor affecting the COVID-19 vaccination programme amongst marginalised groups who already do not engage in health services. Recent evidence supports this, in which a cross-country survey (EU and the UK) found that institutional distrust was a key driver of lower vaccination rates [71]. Surveyed attitudes of minoritised ethnic groups towards the COVID-19 vaccine in the UK cited mistrust towards the government and fears rooted in previous medical malpractice [72]. As the findings in our study show, for female participants from minoritised ethnic backgrounds who experienced side-effects from the vaccines, these side effects embodied the fears around safety and distrust and confirmed pre-existing anxieties. As such, responses to the vaccination programme are interwoven with the context of a history of institutionalised inequalities for marginalised participant groups, which invokes feelings of suspicion and scepticism at the motivations behind the vaccination rollout.

## Conclusions & policy implications

Monitoring attitudes to vaccines remains a relevant and important area for academic research, not only for the continued rollout of the COVID-19 vaccination globally and the need for repeated booster jabs, but for future public health crises, as well as health inequalities exacerbated by the unequal impacts of the pandemic. The findings detailed here show how wider social inequalities, intersecting with experiences of marginalisation and the (re)production of inequalities during the pandemic, has long-lasting and widespread implications vis-à-vis the COVID-19 vaccination programme. More evidence is emerging about the unequal legacy impacts from the pandemic, alongside evidence of illegal gatherings during lockdown by leading policy-makers [73, 74] which threatens to undermine public confidence in centralised institutions. On top of this, many are faced with a cost-of-living crisis, and an unequal response to the recent refugee crises is apparent [69, 75]. It is therefore uncertain what the broader implications may be for public trust at the institutional level. The implications of this may already be apparent, where new data has suggested that MMR vaccination rates are at a ten year low since the start of the pandemic [76], and other childhood immunisations rates have also fallen [77].

This research provides further understanding of the factors surrounding vaccination uptake, for future public health crises and vaccination drives, where public health policy must recognise this broader context. Ongoing research in this space should include contextual factors to better understand and guide the assumptions underpinning public health policy. Prior engagement and information sharing through established networks is crucial.

The themes drawn upon in this article speak to factors that may have broader relevance across forms of research looking at inequalities and public health, related to histories of marginalisation and distrust more generally, which may be particularly relevant in other forms of health and social care research for the assessment of inequalities. More integrated theoretical grounding driven by these themes could be capitalised on in the knowledge production process surrounding health inequalities.

## Data Availability

Data for this research data will not be made publicly available as individual privacy could be compromised. Please contact Stephanie Gillibrand (stephanie.gillibrand@manchester.ac.uk) for further information.

## Footnotes

1 Research for the Future is a NIHR funded online database which members of the public sign up to, to be alerted about opportunities to get involved in research. https://www.researchforthefuture.org/#.

## List of abbreviations

CRAG: Community Research Advisory Group
VCSE: Voluntary, Community and Social Enterprise
